# The cost of rehabilitation after critical illness: a comparison of hospitalization costs for traumatic brain injury and non-traumatic brain injury patients with disorders of consciousness

**DOI:** 10.3389/fpubh.2025.1552162

**Published:** 2025-04-17

**Authors:** Miao Yu, Zhongmou Huang, Yansui Yang, Yulin Wang, Hai Ren, Shilan Tang

**Affiliations:** ^1^School of Healthcare Management, Tsinghua University, Beijing, China; ^2^School of Public Policy and Management, Tsinghua University, Beijing, China; ^3^Institute for Hospital Management, Tsinghua University, Shenzhen, China; ^4^Administrative Office, Shenzhen Longcheng Hospital, Shenzhen, China; ^5^General Management Department, Shenzhen Dapeng New District Medical and Health Group, Shenzhen, China

**Keywords:** hospitalization costs, disorders of consciousness, traumatic brain injury, determinants, China

## Abstract

**Background:**

This study aims to compare hospitalization costs between traumatic brain injury (TBI) and non-traumatic brain injury (non-TBI) patients with disorders of consciousness (DoC) to explore cost determinants.

**Methods:**

A retrospective analysis was conducted on 210 DoC inpatients admitted to Shenzhen Longcheng Hospital, a tertiary rehabilitation hospital located in China’s Pearl River Delta region, between 2015 and 2020. Patients were categorized into TBI (*n* = 44) and non-TBI (*n* = 166) groups based on etiology. Demographic, clinical, and hospitalization cost data were collected for each patient. The study compared the cost composition for DoC patients by etiology and used multivariate analysis to identify factors influencing hospitalization costs.

**Results:**

The median length of stay (LOS) and cost for TBI patients were 363.5 days and $57,366.05, respectively, while for non-TBI patients, the medians were 280.5 days and $57,117.64. Across both groups, the highest cost components were rehabilitation, medication, and treatment expenses. Factors associated with higher hospitalization costs included non-TBI etiology, local residents, medical insurance, LOS, self-employed, surgical treatment, and traditional Chinese medicine (TCM) intervention.

**Conclusion:**

Hospitalization cost structures were similar across etiologies, emphasizing value-driven care priorities. Key factors associated with higher hospitalization costs included non-TBI etiology, local residency, medical insurance, LOS, self-employment status, surgery, and TCM. These findings highlight key drivers of healthcare costs in DoC care, emphasizing the need for targeted policy interventions. However, given the limitations of this study, further research with larger, more diverse samples is essential to comprehensively assess the impact of costs on patient outcomes and care quality.

## 1 Introduction

Intensive rehabilitation, which includes recovery from various types of trauma and severe illness, aims to enhance the physical, psychological, and social functions of critically ill patients. A key focus in this field is the rehabilitation of patients with disorders of consciousness (DoC), a complex condition characterized by different levels of consciousness, including coma, vegetative state, and minimally conscious state. The prevalence of DoC is estimated at 0.2–6.1 per 100,000 people (1, 2), with traumatic brain injury (TBI) and non-traumatic brain injury (non-TBI) as leading causes (3, 4). DoC patients experience high mortality, with rates reaching 30% within 6 months and 50% within 1 year (5, 6). Currently, there are an estimated 100,000–400,000 DoC patients in the United States, with approximately 20,000 patients reported in Europe (7, 8). Estimates suggest that around 300,000 to 500,000 individuals in China are currently living with DoC, despite limited comprehensive epidemiological data. Additionally, over 100,000 new cases are reported each year, contributing to cumulative healthcare costs in the billions of dollars (9–12).

Advances in emergency and intensive care have significantly improved survival rates for DoC patients, with rates now between 40 and 70% (13–15). However, the high demand for long-term rehabilitation has led to increased healthcare costs. In 2010, rehabilitation expenses for DoC patients in the United States totaled $108 billion, representing 0.72% of the gross domestic product (16). Few studies in China have addressed the rehabilitation costs for DoC patients, largely due to high misdiagnosis rates, logistical challenges in conducting field studies, and dispersed treatment settings (7). Understanding the hospitalization costs for DoC patients is crucial for optimizing resource allocation and improving the affordability of long-term care in China’s healthcare system. This study aims to examine differences in hospitalization costs between DoC patients with TBI and non-TBI, as well as to identify factors contributing to these costs. Given the chronic nature of DoC and the substantial financial burden on families and institutions, identifying cost drivers can inform policies to improve affordability and accessibility of long-term care.

## 2 Materials and methods

This study included DoC patients treated at Shenzhen Longcheng Hospital, tertiary rehabilitation hospital located in China’s Pearl River Delta region, from January 2015 to December 2020. Patients with DoC were selected from the electronic medical record system of the hospital, and then reviewed and confirmed by neurologists. Inclusion criteria were (1): DoC patients with consciousness impairment and (2) age 18 years or older. Exclusion criteria were (1) patients under 18 years and (2) patients with incomplete data. Based on these criteria, a total of 210 DoC patients were included, divided into TBI (*n* = 44) and non-TBI (*n* = 166) groups.

### 2.1 Patient and public involvement

Patients or the public were not involved in the design, or conduct, or reporting, or dissemination plans of our research.

### 2.2 Data

We extracted clinical data for 210 DoC patients who were admitted between January 2015 and December 2020 from the hospital’s electronic medical record system. Data collected included age, gender, geographic origin, payment method, marital status, diagnosis category, surgical history, comorbidities, ICU admissions, length of stay (LOS), and hospitalization costs. This retrospective study utilized fully de-identified data and was conducted in collaboration between Tsinghua University and Shenzhen Longcheng Hospital. The joint research project received formal approval and was registered internally by Shenzhen Longcheng Hospital. Following local regulations governing retrospective studies involving anonymized data, the hospital’s ethics committee determined that this study was exempt from further ethical review, as it posed no additional risks to patients and required no interventions. An IRB approval number was not issued due to this exempt status.

### 2.3 Hospitalization costs and categories

**Figure 1:**
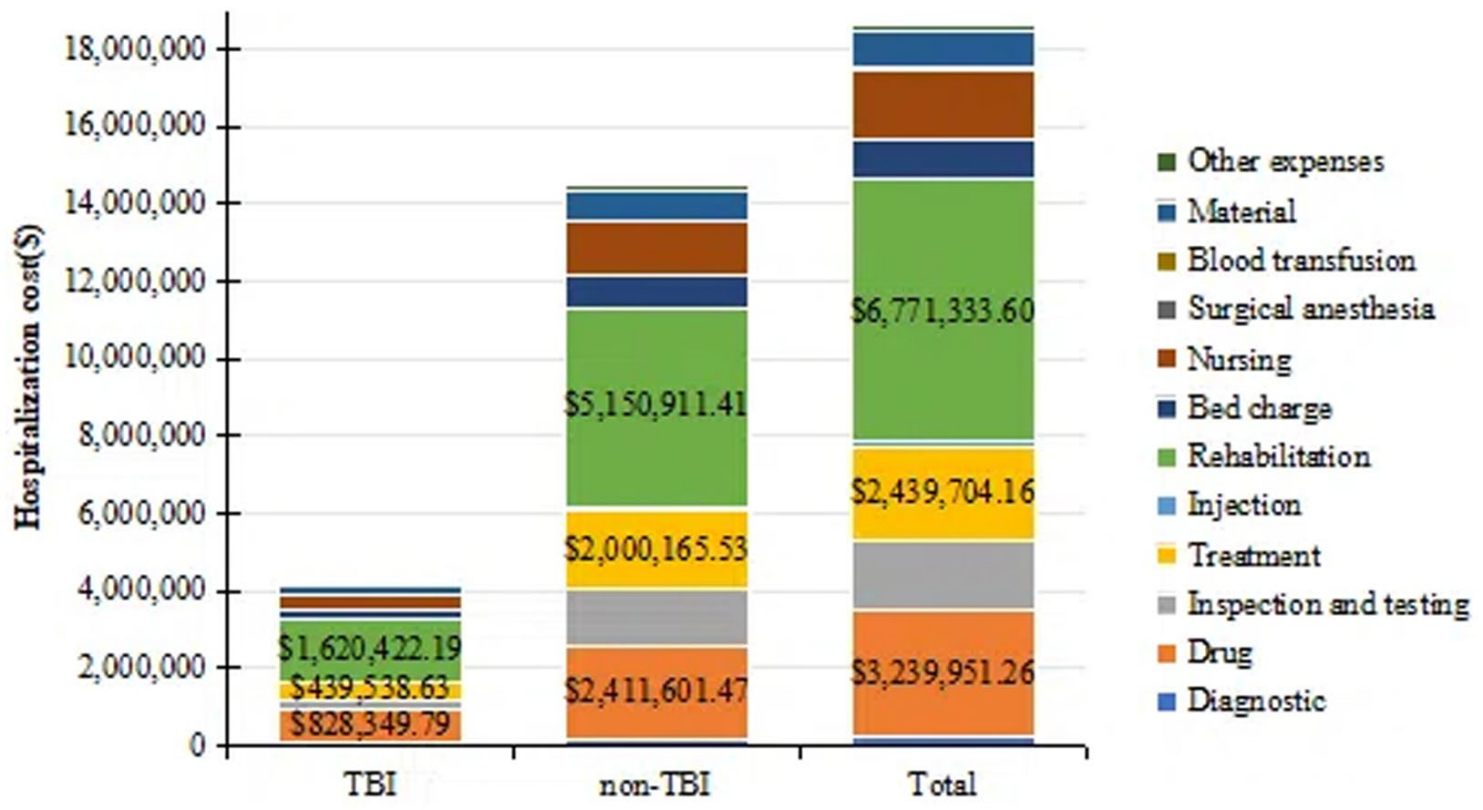
A bar chart showing the total hospitalization costs for two groups of DoC patients and costs for all major categories. TBI, traumatic brain injury. All values are in US dollars (US$).

All patients had continuous hospital stays, with consistently high overall costs. Adjustments for the Consumer Price Index (CPI) showed minimal variation; thus, raw data were used for cost analysis. Costs were converted to U.S. dollars (US$) at an average exchange rate (17) of US$1 = 6.6727 (from 2015 to 2020). Hospitalization costs were categorized as follows (1): diagnostic, (2) drug, (3) inspection and testing, (4) treatment, (5) rehabilitation, (6) injections, (7) bed charge, (8) nursing, (9) surgical anesthesia, (10) blood transfusion, (11) material, and (12) other expenses (Figure 1).

### 2.4 Statistical analysis

Descriptive analyses were performed for continuous and categorical variables, presented as mean ± standard deviation, median (interquartile range, IQR), frequency, and percentage. T-tests or Wilcoxon rank-sum tests were applied based on data distribution. Multivariate analysis was conducted using a multiple linear regression model. Since hospitalization cost data were not normally distributed, a logarithmic transformation (base 10) was applied. Statistical significance was set at *p* < 0.05. All analyses were conducted using IBM SPSS 26.0 (IBM, Armonk, NY, USA).

## 3 Results

### 3.1 Basic information

A total of 210 patients were included in this study, with 44 in the TBI group and 166 in the non-TBI group. The median age for TBI patients was 51.5 years (IQR: 34.25–58.75), while for non-TBI patients, it was 58.0 years (IQR: 50.00–71.00). Males accounted for the majority in both groups (TBI: 72.7%; non-TBI: 71.7%), and most patients were covered by medical insurance (TBI: 70.5%; non-TBI: 94.0%). Geographic data indicated that TBI patients were primarily from out of town (79.5%), whereas non-TBI patients mainly resided locally (54.2%). Comorbidities differed between groups: hypertension was present in 22.7% of TBI patients and 57.8% of non-TBI patients, and diabetes in 4.5% of TBI patients and 24.7% of non-TBI patients. TBI patients had longer median LOS and higher median costs than non-TBI patients. The median LOS for TBI and non-TBI patients was 363.50 days (IQR: 123.25–673.75) and 280.50 days (IQR: 115.25–481.25), respectively, with median hospitalization costs of $57,366.05 (IQR: 21,593.64–137,545.86) for TBI patients and $57,117.64 (IQR: 28,083.32–116,947.80) for non-TBI patients (Table 1).

**Table 1:** Basic characteristics and hospitalization costs of the two groups.

Variables		TBI (*n* = 44)	non-TBI (*n* = 166)	*p*-value
Gender, *n*, %	Male	32 (72.7%)	119 (71.7%)	0.892
	Female	12 (27.3%)	47 (28.3%)	
Local residents, *n*, %	Y	9 (20.5%)	90 (54.2%)	0.000
	N	35 (79.5%)	76 (45.8%)	
Occupations, *n*, %	Urban workers	25 (56.8%)	50 (30.1%)	0.153
	Retirees	1 (2.3%)	22 (13.3%)	
	self-employed	4 (9.1%)	58 (34.9%)	
	Others	14 (31.8%)	36 (21.7%)	
Medical insurance, *n*, %	Y	31 (70.5%)	156 (94.0%)	0.000
	N	13 (29.5%)	10 (6.0%)	
Marital status, *n*, %	Married	34 (77.3%)	140 (84.3%)	0.270
	Others	10 (22.7%)	26 (15.7%)	
Surgery treatment, n, %	Y	29 (65.9%)	126 (75.9%)	0.181
	N	15 (34.1%)	40 (24.1%)	
Hypertension, *n*, %	Y	10 (22.7%)	96 (57.8%)	0.000
	N	34 (77.3%)	70 (42.2%)	
Diabetes, *n*, %	Y	2 (4.5%)	41 (24.7%)	0.003
	N	42 (95.5%)	125 (75.3%)	
ICU, *n*, %	Y	13 (29.5%)	72 (43.4%)	0.097
	N	31 (70.5%)	94 (56.6%)	
TCM, *n*, %	Y	28 (63.6%)	83 (50.0%)	0.108
	N	16 (36.4%)	83 (50.0%)	
Age, year, median (IQR)		51.50 (34.25–58.75)	58.00 (50.00–71.00)	0.000
LOS, day, median (IQR)		363.50 (123.25–673.75)	280.50 (115.25–481.25)	0.306
Number of diagnoses, *n*, %		7.00 (5.25–10.00)	6.00 (1.00–10.00)	0.054
Hospitalization costs, median (IQR)		57,366.05 (21,593.64–137,545.86)	57,117.64 (28,083.32–116,947.8)	0.854

All values are in US dollars (US$). TBI, traumatic brain injury; ICU, intensive care unit; TCM, traditional Chinese medicine; LOS, length of stay.

### 3.2 Hospitalization cost composition

**Figure 2:**
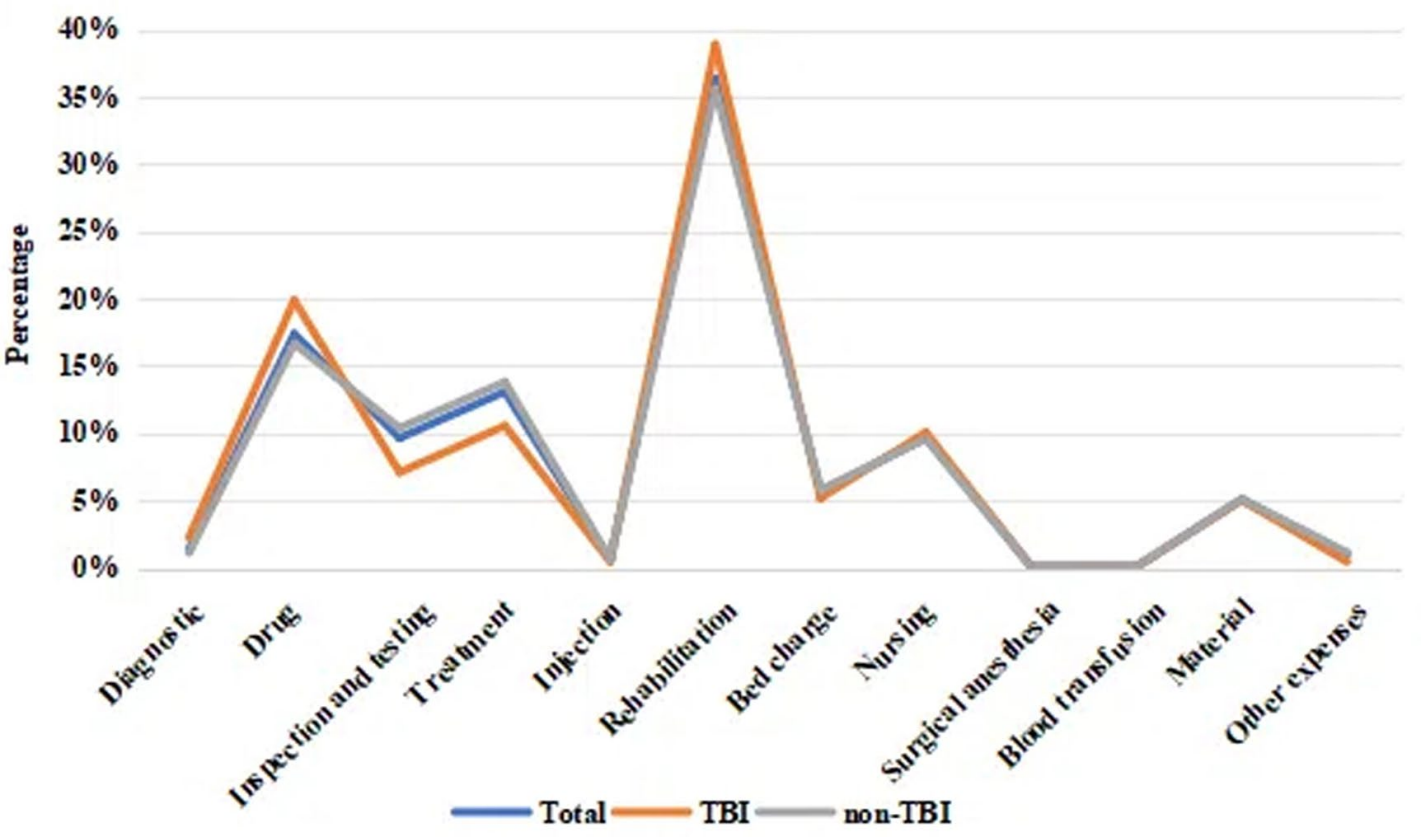
A line chart showing the total costs of hospitalization for DoC patients and the proportion of costs for all major categories. TBI, traumatic brain injury.

Regarding the cost composition (Figure 2, Table 2), the top three categories for both groups were rehabilitation (total: 36.33%, TBI: 38.90%, non-TBI: 35.59%), drugs (total: 17.39%, TBI: 19.89%, non-TBI: 16.66%), and treatment (total: 13.09%, TBI: 10.55%, non-TBI: 13.82%). TBI patients had higher expenditures for diagnostics, drugs, rehabilitation, and bed charges, while non-TBI patients had higher costs for inspection and testing, treatment, and nursing (Table 3). Only diagnostic costs differed significantly (*p* < 0.05), with no significant differences in other categories (*p* > 0.05).

**Table 2:** Proportion of hospitalization costs.

Variables	Diagnostic	Drug	Inspection and testing	Treatment	Injection	Rehabilitation	Bed charge	Nursing	Surgical anesthesia	Blood transfusion	Material	Other expenses
Total (%)	1.38	17.39	9.70	13.09	0.58	36.33	5.65	9.69	0.09	0.08	5.10	1.00
TBI (%)	2.25	19.89	7.09	10.55	0.47	38.90	5.18	10.06	0.09	0.04	5.00	0.40
non-TBI (%)	1.13	16.66	10.39	13.82	0.61	35.59	5.78	9.58	0.09	0.09	5.10	1.10

TBI, traumatic brain injury.

**Table 3:** Comparison of the internal composition of hospitalization costs between the two groups.

Variables	TBI (*n* = 44)	non-TBI (*n* = 166)	*p*-value
Diagnostic	1,084.04 (480.58–2,804.30)	658.13 (250.95–1,095.06)	0.000
Drugs	10,717.59 (21,550.985–212,728.700)	8,158.99 (3,189.14–18,229.36)	0.393
Inspection and testing	16,439.600 (3,229.72–31,880.45)	4,578.55 (1,934.09–10,034.04)	0.063
Treatment	4,734.16 (2,133.53–13,570.20)	6,712.79 (2,481.46–14,305.39)	0.512
Injection	216.61 (80.34–515.81)	259.95 (103.62–658.47)	0.332
Rehabilitation	19,555.17 (6,081.66–68,076.37)	18,292.46 (7,196.86–38,915.35)	0.321
Bed charge	3,087.28 (933.47–7,796.58)	2,899.50 (1,010.61–6,035.79)	0.688
Nursing	4,142.91 (1,614.41–14,846.34)	5,648.18 (2,079.11–12,654.46)	0.892
Surgical anesthesia	11.24 (0.00–87.48)	16.86 (3.75–63.69)	0.652
Blood transfusion	0.00 (0.00)	0.00 (0.00)	0.322
Material	2,073.48 (1,028.35–5,870.67)	2,534.68 (1,007.73–5,955.44)	0.759
Other expenses	237.98 (42.37–735.05)	329.51 (63.02–1,416.41)	0.109

All values are in US dollars (US$). TBI, traumatic brain injury.

### 3.3 Factors influencing hospitalization costs

Multivariate linear regression analysis indicated a significant model (*F* = 34.084, *p* < 0.001). Factors associated with higher hospitalization costs included non-TBI etiology (β = 0.087, *p* < 0.05), local residents (β = 0.109, *p* < 0.05), medical insurance (β = 0.116, *p* < 0.05), LOS (β = 0.590, *p* < 0.001), self-employed individuals (β = 0.124, *p* < 0.05), surgical treatment (β = 0.241, *p* < 0.001), and traditional Chinese medicine (TCM) interventions (β = 0.122, *p* < 0.05). Together, these variables explained 71.7% of the total variance in hospitalization costs (Table 4).

**Table 4:** Multiple linear regression with total hospitalization cost as dependent variable.

Variables	B	SE	β	*t*	*p*	95% CI	*F*	Adjust *R*-square
TBI	0.104	0.053	0.087	1.980	0.049	0.000, 0.208	34.084	0.717
Gender	0.011	0.044	0.010	0.243	0.808	−0.076, 0.098		
Age	0.000	0.002	−0.009	−0.165	0.869	−0.004, 0.003		
Local residents	0.106	0.044	0.109	2.397	0.017	0.019, 0.194		
Occupations								
Retirees	0.000	0.066	0.000	0.001	0.999	−0.130, 0.130		
self-employed	0.132	0.065	0.124	2.039	0.043	0.004, 0.261		
Others	−0.079	0.053	−0.069	−1.499	0.135	−0.184, 0.025		
Medical insurance	0.182	0.065	0.116	2.813	0.005	0.054, 0.310		
Marital status	0.015	0.052	0.012	0.293	0.770	−0.087, 0.117		
LOS	0.001	0.000	0.590	13.867	0.000	0.001, 0.001		
Surgery treatment	0.268	0.045	0.241	5.954	0.000	0.179, 0.357		
Hypertension	−0.024	0.043	−0.025	−0.558	0.577	−0.109, 0.061		
Diabetes	−0.067	0.051	−0.055	−1.294	0.197	−0.168, 0.035		
Number of diagnoses	−0.006	0.005	−0.055	−1.379	0.169	−0.015, 0.003		
ICU	0.069	0.041	0.070	1.673	0.096	−0.012, 0.151		
TCM	0.120	0.041	0.122	2.940	0.004	0.039, 0.200		
Constant	4.786	0.129		37.240	0.000	4.533, 5.040		

TBI, traumatic brain injury; ICU, intensive care unit; TCM, traditional Chinese medicine; LOS, length of stay.

## 4 Discussion

Quantifying hospitalization costs for DoC patients is essential for optimizing resource allocation and informing policy decisions. This analysis enables healthcare systems to design equitable insurance frameworks, reduce catastrophic out-of-pocket expenses (especially in underinsured regions), and ensure economically sustainable care delivery. Simultaneously, it supports clinical quality assessment, provides objective benchmarks for evaluating treatment efficacy, and guides targeted counseling for patients and families on financial planning, while informing the creation of tailored financial support programs. This study addresses a significant gap in research by analyzing the hospitalization costs for DoC patients in China. Specifically, we examined cost differences and identified the determinants for hospitalization expenses among DoC patients with traumatic and non-traumatic etiologies. These findings provide valuable insights to inform policy and resource allocation strategies aimed at optimizing healthcare delivery and reducing medical costs for this patient population.

### 4.1 Etiology and demographic characteristics

The etiology of DoC is varied, with TBI widely recognized as a major cause (18); however, non-TBI cases also play a crucial role. Consistent with prior research, our study found that DoC predominantly affects male patients across both etiologies (TBI: 72.7%; non-TBI: 71.7%), and that TBI patients tend to be younger than non-TBI patients. This demographic distribution aligns with similar studies on DoC patient characteristics in developing countries, suggesting a comparable epidemiological pattern (19). In terms of cost composition, hospitalization expenses in both TBI and non-TBI groups showed similar trends, with rehabilitation costs constituting the largest component, followed by drug and treatment expenses. This cost distribution aligns with healthcare expenditure trends in developed nations, underscoring the critical focus on rehabilitation in DoC patient management (7, 20). Our study found that rehabilitation accounted for approximately 30% of the total hospitalization costs, which is consistent with existing research and highlights the centrality of rehabilitation in DoC care (7, 8).

### 4.2 Medical costs across healthcare systems

Annual costs for a single DoC patient range from $120,000 to $180,000, with lifetime care expenses exceeding $1,000,000 (21). However, care costs vary significantly across healthcare systems. For instance, the United States employs a market-driven hybrid model reliant on private providers for advanced services and partial policy support. However, insurance gaps and stringent asset assessments often leave low-income families vulnerable to catastrophic bills, creating a ‘high-resource, high-burden, low-equity’ dilemma. Nordic countries (e.g., Sweden, Norway) adopt universal welfare models funded by high taxation, where public institutions deliver specialized care at minimal out-of-pocket costs. While this ensures ‘high-equity, high-cost, low-flexibility,’ it sacrifices technological innovation and access to premium services (22, 23). In contrast, China’s social insurance system, anchored by public hospitals, provides broad basic coverage (50–70% reimbursement) but struggles with long-term care reliance on families, regional disparities, and insufficient commercial insurance supplementation, resulting in a ‘wide-coverage, low-cost, weak-sustainability’ framework. Despite higher baseline accessibility, China’s long-term care system and urban–rural equity require urgent improvement.

### 4.3 Influencing factors of medical costs

Our analysis identified several key factors influencing hospitalization costs in DoC patients. Etiology emerged as a significant determinant. Non-TBI patients incurred higher costs, which may be attributed to greater disease complexity and prognostic uncertainty compared to TBI patients (24). Regional differences emerged as a notable factor, with local patients incurring higher total hospitalization costs compared to non-local patients. This disparity may be attributed to economic factors, as local patients reside in economically developed cities in eastern China, where higher disposable incomes and greater accessibility to advanced healthcare services drive up hospitalization expenses. Additionally, regional disparities in medical insurance reimbursement policies may contribute to variations in hospital billing practices, further influencing cost differences across regions (20, 25).

Medical insurance was the primary payment method among DoC patients; however, insured patients exhibited higher total hospitalization costs. Insurance coverage may encourage greater utilization of inpatient services, potentially increasing demand and associated hospital costs (26). Additionally, reimbursement structures may influence physician decision-making, sometimes leading to more intensive or costlier treatment approaches (27). Basic medical insurance in China has markedly increased healthcare utilization, particularly for inpatient services (28). However, lower copayment ratios may induce overuse of resources (29). Insurance types differentially influence costs (30): government-funded plans with low copayments risk demand inducement, while private commercial insurance may curb overutilization but exacerbate inequitable resource allocation (31) Even insured families face financial toxicity due to high out-of-pocket expenses for complex conditions like DoC, especially for non-reimbursable long-term care and medications (32). Policy reforms should balance cost containment and household protection through tiered reimbursement schemes, priority coverage for low-income groups, and dynamic monitoring of insurance expenditures. Future research must refine analyses of insurance heterogeneity and address access barriers for vulnerable populations to build an inclusive, cost-effective payment system.

Socioeconomic disparities in healthcare access are significantly associated with delayed medical care-seeking behaviors among low-income populations (33). Self-employed individuals were found to have higher hospitalization costs, likely due to the lack of stable health coverage, which exposes them to greater financial risk in medical expenses, particularly during long-term hospitalization (34, 35). Financial constraints may exacerbate disease progression, ultimately necessitating hospitalization, while inadequate health insurance coverage compounds this burden by impeding timely reimbursement of medical expenditures (36). The association between insurance coverage and higher hospitalization costs suggests a need for policy reforms to balance service utilization with cost containment, such as value-based payment models that incentivize efficient care. LOS was also a crucial factor, with longer stays being associated with higher costs (37). Complete consciousness restoration remains clinically challenging, and most patients instead remain in various states of impaired consciousness (38). The chronic nature of DoC frequently requires prolonged hospital stays. While acute unconsciousness poses substantial clinical challenges, it should not deter continuous rehabilitation efforts focused on restoring consciousness (2, 39). Surgical interventions were associated with higher total hospitalization costs, likely reflecting the immediate increase in expenses from surgery. Nevertheless, surgical treatment may reduce overall long-term costs by improving patient outcomes and reducing future healthcare needs (37). Therapeutic options for DoC remain limited. Medications such as amantadine have demonstrated efficacy in accelerating functional recovery (40). Chinese studies also indicate benefits of traditional interventions like acupuncture and herbal medicine in improving clinical outcomes (41, 42). However, their impact on healthcare costs is not uniformly positive and may lead to higher expenses (43).

### 4.4 Long-term family economic burden

Although we only examined hospitalization costs, the long-term care of patients with DoC presents families with profound economic burdens and systemic social challenges. Households must shoulder direct medical expenses exceeding hundreds of thousands of yuan annually, including costs for ventilator support, enteral nutrition, and specialized rehabilitation, compounded by non-reimbursable expenditures such as imported medical supplies and transitional ICU care that rapidly deplete family savings (44). Indirect costs further strain families as primary caregivers are often forced to leave the workforce or reduce employment to part-time, resulting in significant income loss, while additional hidden expenses accrue from assistive devices, home modifications, and hired caregivers (45). These financial pressures are exacerbated by structural limitations in China’s medical and long-term care insurance systems, particularly in remote areas where inadequate coverage and reimbursement rates intersect with scarce medical resources and limited income sources, pushing many families into therapeutic poverty. The cumulative effect of these economic and caregiving demands disrupts family dynamics, increasing caregivers’ vulnerability to chronic fatigue, anxiety, depression, and cognitive decline, while the breakdown of social support networks fosters emotional isolation and familial tension that erodes overall quality of life (46, 47). Addressing this complex crisis requires a comprehensive, phased approach: immediate action to include DoC patients in public long-term care insurance with clear reimbursement standards; intermediate steps to develop income-based medical assistance, community respite services, and telemedicine networks; and long-term investments in smart assistive technologies and caregiver training, supported by tax incentives and flexible workplace policies. Ultimately, a sustainable solution demands government leadership, market participation, and community collaboration to equitably distribute medical-economic risks and restore family and social stability.

### 4.5 Recommendations for policy optimization

Over the past three decades, China has made significant progress in rehabilitation services with health insurance policies serving as a critical driver in promoting rehabilitation service utilization (48). Despite progressive expansion of insurance coverage, the sustainability of medical insurance funds remains a pressing challenge, particularly in regions with developing healthcare infrastructure where inadequate insurance coverage for rehabilitation programs persists (49). This coverage gap may constrain functional recovery, diminish quality of life, and escalate long-term healthcare costs, thereby highlighting the urgent unmet needs for equitable rehabilitation services in these areas.

The transition from the acute to subacute phase represents a critical vulnerability in the continuum of care for patients with DoC. Unlike China’s longstanding gaps in subacute care, the United States and the United Kingdom have established robust subacute care models. In these systems, after acute-phase treatment, patients can transition to appropriate medical institutions based on clinical needs, supported by bidirectional referral mechanisms, clear functional differentiation among facilities, and streamlined care pathways. These countries utilize validated assessment tools to rapidly evaluate rehabilitation progress, enabling tailored interventions encompassing supportive therapy, rehabilitation, nursing, and daily living assistance.For instance, the U.S. Post-Acute Care (PAC) allows patients to access diverse care settings, including skilled nursing facilities, home health agencies, long-term care hospitals, and inpatient rehabilitation centers, addressing complex care needs. Similarly, the U.K. Intermediate Care (IC) model offers flexible options such as hospital-at-home services, geriatric day wards, nurse-led units, community hospitals, and residential care homes. In contrast, China has yet to develop a comprehensive three-tiered rehabilitation system. Patients with severe conditions like DoC often face care discontinuity during the acute-to-subacute transition. Currently, China is actively constructing an integrated care framework to bridge gaps between rehabilitation stages, with the goal of refining its rehabilitation system to better meet the needs of DoC patients (10).

The “Healthy China 2030” strategic plan prioritizes health as a strategic cornerstone of national development, with rehabilitation playing a pivotal role in improving the population’s overall functional capacity and quality of life. This strategy is grounded in the principles of “shared participation and universal health,” calling for the expansion of rehabilitation services to reach broader demographics and address their escalating needs (50). The findings provide actionable insights for China’s healthcare reform agenda under the Healthy China 2030 strategic framework, advocating three synergistic measures: extending insurance benefits to comprehensive rehabilitation services, implementing standardized reimbursement protocols to mitigate interprovincial cost differentials, and prioritizing infrastructure development in central and western regions through risk-adjusted capitation models. Persistent regional disparities in care delivery further necessitate targeted fiscal allocations to achieve service parity, ensuring equitable resource distribution while aligning payment reforms with clinical value imperatives.

There are limitations to our study. First, the relatively small sample size may affect the generalizability of our findings. Larger studies are necessary to confirm our results and enhance understanding of cost determinants in DoC care. Second, some potential factors affecting hospitalization costs, such as patient income, were not included. Third, TBI constituted approximately 20% of our sample, potentially limiting insights into the cost implications for TBI patients specifically. Future research should include a larger proportion of TBI cases to deepen understanding in this area. Fourth, while this study quantifies hospitalization costs, it does not explore the correlations between these costs and critical clinical outcomes, such as Glasgow Outcome Scale scores or mortality rates. Future longitudinal studies should aim to integrate detailed cost data with specific neurofunctional recovery metrics in order to comprehensively evaluate cost-effectiveness and provide robust evidence for value-based care models. Finally, as this study was based on data from a single hospital, the generalizability of findings may be constrained.

In summary, the rehabilitation hospitalization costs for DoC patients are influenced by multiple factors, including the patient’s individual condition, utilization of medical resources, and socioeconomic variables, necessitating comprehensive consideration. Future research should delve into the long-term effects of different treatment approaches and the role of socioeconomic factors in care delivery to optimize rehabilitation protocols, improve efficiency, reduce costs, and ultimately better address patient needs. Furthermore, establishing robust predictive models is essential for evaluating patients’ rehabilitation potential and associated costs, as well as for designing personalized rehabilitation plans.

## 5 Conclusion

This study highlights the substantial economic burden of DoC care in China, driven by prolonged hospitalization and rehabilitation demands. While rehabilitation remains the cornerstone of cost allocation across etiologies, factors such as insurance coverage and regional inequalities further amplify financial pressures. These findings underscore the urgent need for policymakers to prioritize cost-effective strategies within the Healthy China 2030 framework. Immediate actions should include expanding insurance coverage for long-term rehabilitation, standardizing reimbursement protocols to bridge regional gaps, and integrating value-based payment models to align clinical outcomes with fiscal sustainability. For researchers, advancing studies on the cost-effectiveness of rehabilitation interventions and the socioeconomic determinants of care is critical. Future research should prioritize investigating the differences in rehabilitation outcomes and associated costs across different stages of patient recovery, as this will provide valuable insights for optimizing resource allocation and improving long-term prognosis. Addressing these challenges is not merely an economic imperative but a moral obligation to ensure equitable, high-quality care for all DoC patients. The time to act is now—delays will only exacerbate the societal and familial burdens of this vulnerable population.

## Data Availability

The original contributions presented in the study are included in the article/supplementary material, further inquiries can be directed to the corresponding author.
